# Patient-Reported Factors Alleviating Pain Among Persons With Cancer

**DOI:** 10.1093/geroni/igaa057.661

**Published:** 2020-12-16

**Authors:** Dottington Fullwood, Roger Fillingim, Diana Wilkie

**Affiliations:** 1 The University of Florida, Gainesville, Florida, United States; 2 University of Florida, Pain Research and Intervention Center of Excellence, Gainesville, Florida, United States; 3 University of Florida, Gainesville, Florida, United States

## Abstract

Pain impacts wellbeing and is among the most common symptoms of cancer. Factors that decrease pain severity have been understudied despite their importance for high-quality cancer care. The study purpose was to describe pain alleviating factors and their association with type of cancer. This secondary comparative analysis included 579 participants from studies of inpatients and outpatients with cancer (mean age=58.7±12.3; 27.3% female; 85.5% White, 5.7% Black, 7.6% Other). They completed the McGill Pain Questionnaire on paper or a tablet computer. To determine factors that alleviated pain, we focused on the open-ended question: 1) What kinds of things relieve your pain? We coded text responses into six outcome categories: 1) Activity level, 2) Cognitive, 3) Environmental, 4) Medical, 5) Physical, and 6) Sedentary behavior. We counted the number of activities/factors in each category and conducted multivariable regression analysis adjusting for sociodemographic constructs. Adjusted models revealed that activity (ρ=0.02), cognition (ρ<0.001) and medication (ρ<0.001) were more often endorsed as alleviating factors among individuals living with lung cancer compared to head and neck cancer participants. Those diagnosed with lung cancer (ρ=0.02) and males (ρ=0.02) utilized significantly less physical alleviating factors than head and neck cancer individuals and females. This is the first study to examine pain-alleviating factors among individuals living with cancer. These findings contribute new information regarding activities that alleviate pain among cancer survivors. These findings could inform interventions to promote safe, personalized care designed to alleviate cancer-pain.

